# Laparoscopic Management of Cholecystoduodenal and Cholecystocolic Fistula: A Clinical Case Report

**DOI:** 10.7759/cureus.40657

**Published:** 2023-06-19

**Authors:** Milton Alberto Muñoz Leija, Marion Carolina Alemán-Jiménez, Heliodoro Plata-Álvarez, Victor Daniel Cárdenas-Salas, Ramiro Valdez-López

**Affiliations:** 1 General Surgery, Hospital General de Zona 6, Instituto Mexicano del Seguro Social, San Nicolas de los Garza, MEX; 2 Human Anatomy, Universidad Autonoma de Nuevo Leon, School of Medicine, Monterrey, MEX

**Keywords:** subtotal cholecystectomy, laparoscpic surgery, cholecysto-duodenal fistula, biliary enteric fistula, gall blader disease

## Abstract

Biliary fistula is a rare (less than 8%) cholecystectomy complication, internal fistulae being the most common of them (mainly colonic and duodenal). However, the presence of two fistulas at the same time is extremely rare, with a small number of cases reported in the literature to date. Symptoms tend to be non-specific, leading to a difficult preoperative diagnosis. The standard treatment for bilioenteric fistulas is open cholecystectomy and subsequent closure of the fistula. Nonetheless, modern techniques including laparoscopic and endoscopic approaches have been reported lately for their treatment with favorable results. We present a case of concomitant cholecystoduodenal and cholecystocolic fistula successfully treated with subtotal cholecystectomy and primary closure of the fistulous tracts by laparoscopic approach in a female Hispanic patient.

## Introduction

A biliary fistula consists of communication or an abnormal passage from the biliary system to an organ, cavity, or skin. These are classified as internal (bilioenteric, biliobiliary, broncho-biliary) or external (biliary-cutaneous) [1]. Bilioenteric fistulas have been found to be between 1% and 8% of cholecystectomies (laparoscopic and conventional). The cholecystoduodenal fistula is the most common with a prevalence of 70%. The cholecystocolonic fistulae are the second most common with a prevalence of 8%-26% [2-3]. The association of cholecystoduodenal and cholecystocolic fistulae is an extremely rare observation, with around 20 cases reported in the literature up to date [4]. The etiology is related to the presence of malignancy of the biliary tract, Crohn’s disease, ulcerative colitis, parasitosis, or diverticular disease. Yet, more than 60% of cases are a complication of gallbladder stones [4-5]. Symptoms are non-specific and include abdominal pain, fever, nausea, flatulence, bowel obstruction, fat intolerance, and diarrhea [6]. The standard treatment of bilioenteric fistula is open cholecystectomy and subsequent closure of the fistula [7], however, modern techniques are changing this management according to the surgeon‘s expertise, the conditions of the patient, and the resources availability [3].

This article reports a rare case of cholecystoduodenal and cholecystocolic fistula successfully treated with subtotal cholecystectomy and primary closures of both fistulas by laparoscopic approach.

## Case presentation

A 50-year-old Hispanic female patient with a medical history of diabetes mellitus type II and hypertension presented to the general surgery outpatient clinic with a two-month history of recurrent abdominal pain in the right quadrant, exacerbated by copious meal intake. Nausea, vomiting, average bowel movement frequency change, fever, jaundice, or hematemesis were denied. Physical examination was negative for Murphy’s sign and rebound tenderness. Laboratory workup revealed: white blood cell count 7.36 x 109/L, hemoglobin 12.9 g/dL, aspartate aminotransferase (AST) 18.3 U/L, alanine transaminase (ALT) 16 U/L, alkaline phosphatase 52 U/L, total bilirubin 0.3 mg/dL, and direct bilirubin 0.14 mg/dL. Upon upper abdominal ultrasound, liver and intrahepatic duct were identified without any abnormal findings; gall bladder was identified revealing dense posterior acoustic shadowing and thickness of the wall (3 mm). The common bile duct was normal, measuring 4 mm in diameter. 

**Figure 1:**
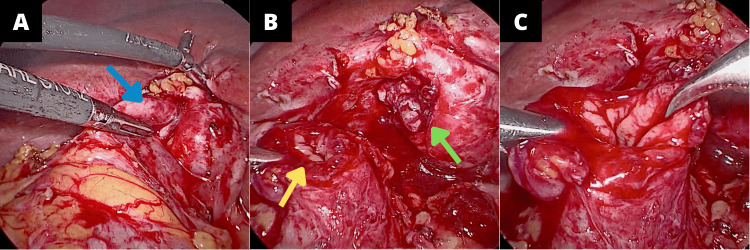
Laparoscopic view of the gallbladder and transverse colon. (A) Cholecystocolic fistula (blue arrow). (B) View of the colon (yellow arrow) and gallbladder (green arrow) with the resected fistula. (C) Colon after fistula resection.

Elective surgery with laparoscopic cholecystectomy was decided. Intraoperative findings revealed a gallbladder adhered to the surrounding omentum and transverse colon, with a fistulous communication of the fundus to the transverse colon (Figure 1).

**Figure 2:**
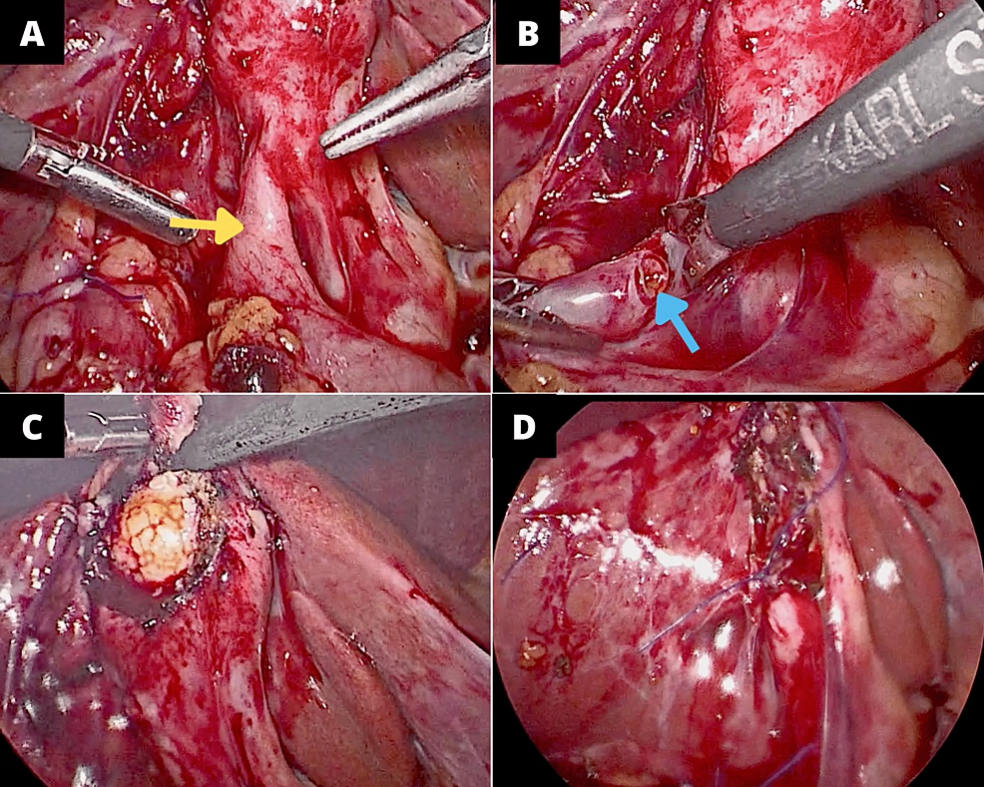
Laparoscopic view of the gallbladder. (A) Cholecystoduodenal fistula (yellow arrow). (B) View of the duodenum (blue arrow) and gallbladder with the resected fistula. (C) Stone being removed from the fundus of the gallbladder. (D) Closure after partial cholecystectomy.

Gallbladder and transverse colon were dissected, and the fistula was excised. The remaining defect of the colon was repaired using a continuous absorbable suture and a non-absorbable suture as a second layer. After gallbladder mobilization, a second fistula was found between Hartmann’s Pouch and the second part of the duodenum (Figure 2A,B). The fistulous tract was excised, and the duodenum defect was then sutured in two layers as performed in the colon. Subsequently, partial cholecystectomy was performed due to the adherence between the common bile duct, gallbladder, and duodenum. A 3-cm stone was removed from the fundus and then closed by an interrupted simple suture (Figure 2C,D). An open drainage was placed in the gallbladder fossa.

The postoperative period was uneventful, with an average serohematic drainage of 10-15 cc per day. Methylene blue leak test was performed on postoperative day five with a negative result, thus oral diet was initialized. On postoperative day seven, the patient was discharged due to an adequate evolution. Drainage was removed after one week, and after one-month laboratory workup and histopathology reports were normal without any evidence of malignancy. 

## Discussion

Cholelithiasis is one of the most frequent pathologies worldwide, accounting for approximately 20 million people, from which 1.8 million present a condition that requires surgery. Cholecystectomy is the most common elective abdominal surgery, with an estimated of 750,000 surgeries performed every year [8]. Bilioenteric fistula is a rare complication of cholelithiasis, with an incidence of 0.15% to 8% [3]. Cholecystoduodenal fistulas are the most common among this pathology (70-80%), leaving cholecystocolonic fistulas next in order (10-20%). The presence of both is extremely rare, and only about 20 cases have been reported in the literature [4]. The rarity of this condition, the difficulty of its diagnosis, under reported cases and the low and middle income countries barriers, may be the rationale behind the small number of papers [9]. 

The standard treatment of a bilioenteric fistula is open cholecystectomy and closure of the fistula [7]. Given to the technical evolution in laparoscopy, the progressive learning curve has led surgeons continually to proficiency in higher levels, and prior situations that would be indicative of laparotomy, now become increasingly amenable in laparoscopic treatment; the treatment of fistulas have also this tendency, not being contraindicated any more to laparoscopy [4,10-11]. However, the intense tissue inflammation can cause the conversion to laparotomy in laparoscopic cholecystectomy procedure [12-13]. In addition, treatment with subtotal cholecystectomy in difficult scenarios has been widely accepted in the literature [14-17]. Management of double bilioenteric fistulas by laparoscopic approach with subtotal cholecystectomy might be an adequate option for surgeons. The use of drainage is recommended in these cases [14].

Some other treatment options recently reported for the closure of both fistulas (colonic and duodenal) include metal stent on the colon, and polyglycolic acid sheets in the duodenum by endoscopic closure [18]. Patient conditions, availability of resources, and expertise of the surgeon and his team are the key factors that will influence the decision of the best treatment option. More studies (case reports, case series or a systematic review) are still needed to evaluate which type of treatment could be considered the gold standard. 

To the best of our knowledge, this is the first case of cholecystoduodenal and cholecystocolic fistulae reported in our country treated successfully by laparoscopic approach.

## Conclusions

Bilioenteric fistulas are a rare presentation of biliary pathology. Treatment of choice is conventional surgery, nonetheless, with the increasing availability of modern techniques such as laparoscopy, this can become an option. Subtotal cholecystectomy plus primary closure of the fistula tract may be an option in complicated cases. More studies are needed to further assess this technique’s effectiveness in treating gallbladder double fistula.

## References

[1] Safaie-Shirazi S, Zike WL, Printen KJ (1973). Spontaneous enterobiliary fistulas. Surg Gynecol Obstet.

[2] Balent E, Plackett TP, Lin-Hurtubise K (2012). Cholecystocolonic fistula. Hawaii J Med Public Health.

[3] Bhat GA, Jain R, Lal P (2016). Cholecystoduodenocolic fistula: an unexpected intraoperative finding, a surgical challenge. Int J Clin Med.

[4] Costi R, Randone B, Violi V (2009). Cholecystocolonic fistula: facts and myths. A review of the 231 published cases. J Hepatobiliary Pancreat Surg.

[5] Cao Y, Li C, Qiao Z (2021). Laparoscopic diagnosis and treatment of cholecysto-colonic fistula: a case report. Asian J Surg.

[6] LeBlanc KA, Barr LH, Rush BM (1983). Spontaneous biliary enteric fistulas. South Med J.

[7] Correia MF, Amonkar DP, Nayak SV (2009). Cholecystocolic fistula: a diagnostic enigma. Saudi J Gastroenterol.

[8] Alexander HC, Bartlett AS, Wells CI (2018). Reporting of complications after laparoscopic cholecystectomy: a systematic review. HPB (Oxford).

[9] Quiroga-Garza A, Garza-Cisneros AN, Elizondo-Omaña RE (2022). Research barriers in the Global South: Mexico. J Glob Health.

[10] Conde LM, Tavares PM, Quintes JL (2014). Laparoscopic management of cholecystocolic fistula. Arq Bras Cir Dig.

[11] Angrisani L, Corcione F, Tartaglia A (2001). Cholecystoenteric fistula (CF) is not a contraindication for laparoscopic surgery. Surg Endosc.

[12] Genc V, Sulaimanov M, Cipe G (2011). What necessitates the conversion to open cholecystectomy? A retrospective analysis of 5164 consecutive laparoscopic operations. Clinics (Sao Paulo).

[13] Chowbey PK, Bandyopadhyay SK, Sharma A (2006). Laparoscopic management of cholecystoenteric fistulas. J Laparoendosc Adv Surg Tech A.

[14] Toro A, Teodoro M, Khan M (2021). Subtotal cholecystectomy for difficult acute cholecystitis: how to finalize safely by laparoscopy-a systematic review. World J Emerg Surg.

[15] Jeong IO, Kim JY, Choe YM (2011). Efficacy and feasibility of laparoscopic subtotal cholecystectomy for acute cholecystitis. Kor J Hepatobiliary Pancreat Surg.

[16] Kulen F, Tihan D, Duman U (2016). Laparoscopic partial cholecystectomy: a safe and effective alternative surgical technique in "difficult cholecystectomies". Ulus Cerrahi Derg.

[17] Beldi G, Glättli A (2003). Laparoscopic subtotal cholecystectomy for severe cholecystitis. Surg Endosc.

[18] Sato S, Chinda D, Tanaka Y (2021). Effective endoscopic closure of cholecysto-duodenal and transverse colon fistulas due to squamous cell carcinoma of the gallbladder using polyglycolic acid sheets and a covered metal stent. Intern Med.

